# Paraventricular Nucleus Infusion of Oligomeric Proantho Cyanidins Improves Renovascular Hypertension

**DOI:** 10.3389/fnins.2021.642015

**Published:** 2021-03-04

**Authors:** Xiao-Jing Yu, Guo-Rui Xin, Kai-Li Liu, Xiao-Jing Liu, Li-Yan Fu, Jie Qi, Kai B. Kang, Ting-Ting Meng, Qiu-Yue Yi, Ying Li, Yao-Jun Sun, Yu-Ming Kang

**Affiliations:** ^1^Department of Physiology and Pathophysiology, Xi’an Jiaotong University School of Basic Medical Sciences, Shaanxi Engineering and Research Center of Vaccine, Key Laboratory of Environment and Genes Related to Diseases of Education Ministry of China, Xi’an, China; ^2^Key Laboratory of Cellular Physiology at Shanxi Medical University, Ministry of Education, Department of Physiology, Shanxi Medical University, Taiyuan, China; ^3^Department of Cardiology, The Second Clinical Medical College, Shanxi Medical University, Taiyuan, China; ^4^Department of Ophthalmology and Visual Sciences, University of Illinois at Chicago, Chicago, IL, United States; ^5^Department of Physiology and Pathophysiology, Xi’an Jiaotong University, Xi’an, China

**Keywords:** hypertension, hypothalamic paraventricular nucleus, oligomeric proantho cyanidins, oxidative stress, neurotransmitters

## Abstract

Oxidative stress plays an important role in the pathogenesis of hypertension. Oligomeric proantho cyanidins (OPC) is the main polyphenol presents in grape seed and is known for its potent antioxidant and anti-inflammatory properties. In the present study, we hypothesize that OPC can attenuate oxidative stress in the paraventricular nucleus of hypothalamus (PVN), ameliorate neurotransmitter imbalance, decrease the blood pressure and sympathetic activity in renovascular hypertensive rats. After induction of renovascular hypertension by the two-kidney one-clip (2K-1C) method, male Sprague-Dawley rats received chronic bilateral PVN infusion of OPC (20 μg/h) or vehicle via osmotic minipump for 4 weeks. We found that hypertension induced by 2K-1C was associated with the production of reactive oxygen species (ROS) in the PVN. Infusion of OPC in the PVN significantly reduced the systolic blood pressure and norepinephrine in plasma of 2K-1C rats. In addition, PVN infusion of OPC decreased the level of ROS and the expression of stress-related nicotinamide adenine dinucleotide phosphate (NADPH) oxidases subunit NOX4, increased the levels of nuclear factor E2-related factor 2 (Nrf2) and antioxidant enzyme, balanced the content of cytokines, increased expression of glutamic acid decarboxylase and decreased the expression of tyrosine hydroxylase in the PVN of 2K-1C rats. Our findings provided strong evidence that PVN infusion of OPC inhibited the progression of renovascular hypertension through its potent anti-oxidative and anti-inflammatory function in the PVN.

## Introduction

Multiple factors contribute to the pathogenesis of hypertension, for instance sympathetic hyperactivity, sensitization of renin–angiotensin systems, and in the molecular level, anomalous signal of G protein-coupled receptor, ROS and the production of pro-inflammatory cytokines (Touyz, 2005; Belmonte and Blaxall, 2011; Muller et al., 2011; Savoia et al., 2011; Zubcevic et al., 2011). ROS plays a crucial part in the pathophysiology of hypertensive disease (Touyz, 2004; Vaziri, 2004; Wilcox, 2005; Rodrigo et al., 2011; Baylis, 2012). Oxidative stress occurs when the generation of ROS and the antioxidant defense systems are unbalanced, which leads to production of inflammatory cytokines including tumor necrosis factor-α, interleukin-6, interleukin-1β (IL-1β), and monocyte chemoattractant protein-1.

Abnormal generation of ROS induces oxidative stress, which thereby cause cellular injury or cytotoxicity (Kakihana et al., 2012; Laurindo et al., 2012; Lee et al., 2012). NADPH oxidases are associated with ROS synthesis in cardio-cerebral-vascular system (Lee et al., 2012). NADPH oxidases-derived ROS rises in hypertensive rats (Wilcox, 2005). Excessive generation of ROS is responsible for depression of nitric oxide bioavailability and antioxidant capacity in the kidney, which exacerbates the pathophysiology of hypertension. Accordingly, the key point of ROS has been implicated in hypertension, as a result of growing generation of ROS in the cardiovascular and cerebrovascular systems (Touyz and Schiffrin, 2004; Vaziri and Rodríguez-Iturbe, 2006; Touyz and Briones, 2011). The PVN, accounting for only about 1% of the cerebrum volume, exerts a vital role in monitoring cardiovascular homeostasis and is a primary region to regulate blood pressure in coordination with neural signals (Badoer, 2001; Ferguson et al., 2008). Oxidative stress in the PVN has been shown to relate to renovascular hypertension (Oliveira-Sales et al., 2009), for which the concrete brain mechanism remains unclarified.

Oligomeric proantho cyanidins (OPC) is one of the polyphenols extracted from grape seeds. It is the oligomeric form of proantho cyanidins with a lower level of polymerization found in food stuffs such as grape seeds and blackberries. Recent studies demonstrated that OPC has a series of pharmacological effects. It can regulate glucose metabolism mainly through protecting pancreatic β-cells by attenuating oxidative stress. it can extend the life span of senescence-accelerated mouse prone/8, a murine model of accelerated senescence, with a slight upregulation of SIRT1, which is recognized as an essential factor for life span extension in the brain (Yokozawa et al., 2011; Sun et al., 2016). It is also shown that OPC has the function of myocardial protection and vasorelaxation by inducing a range of key cellular antioxidant enzymes in cardiovascular cells for its antioxidant activities, free radical-scavenging activities, and lipid peroxidation inhibition. It increases paraoxonase activity, mostly through increasing high-density lipoprotein cholesterol and apolipoprotein A-I levels. OPC may selectively protect against oxidative stress, maintain genomic integrity, and prevent programmed cell death *in vivo* (Lu et al., 2018; Zhu et al., 2018). OPC is reported to be one of the most effective natural antioxidants to scavenge free radicals inside human body. Studies have shown that procyanidins administration in hypertensive rats by oral administration, intragastric administration, etc., can lower blood pressure and affect the expression of molecules or proteins in the central nervous system (Magos et al., 2008). The research related to procyanidins mostly focused in the periphery, and only few studied the mechanism of its function in the central. Therefore, it is important to study the central mechanism of OPC in the treatment of hypertensive disease. Our laboratory and others (Zimmerman et al., 2004; Kang et al., 2004; Han et al., 2011; Su et al., 2014) have found that ROS and pro-inflammatory cytokines in the PVN are related to the regulation of sympathetic nervous activity and blood pressure. To better disinter and display the protective effects of OPC in hypertension, we explored the effects of PVN infusion of OPC on oxidative stress, inflammation and neurotransmitters in hypertensive rats.

Thus, we hypothesize that PVN infusion of OPC inhibits the progression of renovascular hypertension by attenuating oxidative stress in the PVN, ameliorating neurotransmitter imbalance, and thus decreasing the blood pressure and sympathetic activity.

## Materials and Methods

### Animals

Experiments were performed on seven-week-old male Sprague-Dawley rats weighing between 180 and 220 g. The rats were housed with a 12 h light/dark cycle and *ad libitum* access to standard rat chow and water (Zhu et al., 2009; Qi et al., 2019). The experiments were approved by the Animal Care and Use Committee of Xi’an Jiaotong University and conformed to the Guidelines for the Care and Use of Experimental Animals of the United States National Institutes of Health (NIH Publication No. 85-23, revised 1996). All the surgeries were performed under anesthetic and aseptic conditions.

### General Experimental Protocol

Rats were divided into four groups (*n* = 6 per group): SHAM + PVN aCSF, SHAM + PVN OPC, 2K-1C + PVN aCSF, 2K-1C + PVN OPC. Rats had a week of recovery after implantation of bilateral PVN cannula. Baseline blood pressure were measured once per day for 3 days. After that, renovascular hypertensive rats were made by 2K-1C method (Wu et al., 2012; De Kloet et al., 2013). The renal arteries of the sham-operated (SHAM) rats were not occluded. Except that, all surgery procedure was in the same manner. The osmotic minipumps (the rate was 0.11 μL/h, Alzet Model 1004, Durect Corporation, Cupertino, CA, United States) were attached to the PVN cannula for infusion of OPC (solvent: artificial cerebrospinal fluid (aCSF; Sigma, St. Louis, MO, United States), concentration: 20 μg/0.11μl, volume: 0.11μl/h) or aCSF into the PVN for 4 weeks (Kang et al., 2009; Qi et al., 2013). Another set of 2K-1C rats and SHAM rats were treated with a 24h continuous intraperitoneal (IP) infusion of 20 μg/h OPC, or vehicle (normal saline) for 4 weeks with the osmotic minipumps. In the end, rats were euthanized to gather PVN tissues and blood samples for molecular and immunohistochemical views. Some of them were anesthetized for electrophysiological research. To determine the sufficient dose of OPC for PVN infusion, we performed pilot experiment with gradient tests. The current dose (20 μg/h) was the smallest dose which can attenuate the blood pressure in 2K-1C rats.

### PVN Cannula Implantation for Bilateral PVN Infusion

Implantation of bilateral PVN cannula were as mentioned earlier (Gao et al., 2005; Li et al., 2014). An osmotic minipump containing the OPC or aCSF was implanted in the back of neck to attach to the PVN cannula. After the rat was anesthetized with a ketamine (80 mg/kg) and xylazine (10 mg/kg) mixture (i.p.), the head was placed into a stereotaxic apparatus. The skull was then opened, and stainless steel double cannula were implanted into the PVN 1.8 mm posterior, 0.4 mm lateral to the bregma and 7.9 mm ventral to the zero level according to stereotaxic coordinates. The cannula were fixed to the cranium using dental acrylic and two stainless steel screws (Kang et al., 2006). An osmotic minipump filled with the OPC or aCSF was implanted subcutaneously in the back of neck and connected to the PVN cannula. The PVN infusion continued for 4 weeks. Rats received buprenorphine (0.01 mg/kg, s.c.) immediately following surgery and 12 h post operatively. At the end of the experiment, brains were sectioned to verify location of cannula, and only rats with verifiable bilateral PVN injection sites were used in the final analysis. The success rate is about 64%.

### Measurement of Systolic Blood Pressure (SBP)

Systolic Blood Pressure was gauged by a tail-cuff occlusion and acute experiment method. Un-anesthetized rats were warmed to an ambient temperature of 32°C by placing rats in a holding device mounted on a thermostatically controlled warming plate. Rats were allowed to habituate to this procedure for 3 days prior to each experiment. SBP values were averaged from seven consecutive cycles per day obtained from each rat. At the end of the experiment, rats were anesthetized with a ketamine (80 mg/kg) and xylazine (10 mg/kg) mixture (i.p.) and placed dorsally on a heated surgical table. An incision along the blood vessels was made in the thigh near groin, and femoral artery was isolated; polyethylene catheters were placed into the femoral artery and advanced into the abdominal aorta for the measurement of SBP. The catheters, filled with 0.1 ml heparin saline (50 units/ml), were connected to a pressure transducer attached to a digital BP monitor and a polygraph. After waiting for 10 min, SBP data were collected for 20 min and averaged.

### Collection of Blood and Brain Tissue Specimens

Rats were decapitated under anesthesia to gather brain and blood tissue. Trunk blood samples were collected in chilled ethylenediaminetetraacetic acid tubes. Plasma samples were separated and stored at −80°C until assayed. The brain specimens were also reserved at −80°C for later analysis.

### Microdissection of Tissue

The procedure of Tissue microdissection was previously described to separate the PVN (Agarwal et al., 2011). Briefly, the brain was sectioned serially in 300 mm increments from the bregma to lambda using a cryostat. The sections were transferred to coverslip and placed on a cold stage maintained at −10°C. The PVN was punched with the help of a stereotaxic atlas. Some of the microdissected PVN tissue was stored at −80°C until analyzed. The tissues were collected from both sides of the PVN of individual rat.

### Biochemical Assays

The level of norepinephrine (NE) in plasma and IL-10, IL-1β in the PVN of the rats were measured by means of ELISA kits (Biosource International Inc., Camarillo, California). According to the manufacturer’s descriptions, the standards or sample diluents were added in the appropriate well of microtiter plate precoated with specific antibodies and incubated. Conjugate was added and incubated at 37°C for 1 h and then washed. The reactions were stopped with stop solution and read at 450 nm for IL-1β, IL-10, NE measurements using a microtiter plate reader (MK3, Thermo Fisher Scientific, United States).

### Immunohistochemical and Immunofluorescent Studies

Rats were anesthetized and fixed with perfusion using 0.01 M phosphate-buffered solution (PBS) into the left ventricle first and then with 4% paraformaldehyde. The brain samples were collected and soaked in 4% paraformaldehyde and then 30% sucrose. Tissue microdissection was used to separate the PVN tissue. Immunohistochemical and immunofluorescent studies were performed in floating sections as mentioned before (Zimmerman et al., 2004; Kang et al., 2006; Li et al., 2014; Li et al., 2020) to reveal IL-10, IL-1β, Fra-LI, TH, NOX4 expressions in the PVN. Sections were then washed in PBS for 20 min, permeabilized in 0.2% Triton in Tris-buffered saline for 1 h, blocked using 5% normal goat serum with 0.2% Triton in Tris-buffered saline for 1 h, and incubated with primary antibody in blocking buffer at 4°C overnight. The primary antibodies used were: IL-10 (sc-1783, 1:100 dilution), IL-1β (sc-1251, 1:100 dilution), Fra-LI (sc-253, 1:100 dilution), tyrosine hydroxylase (TH) (sc-25269, 1:100 dilution), NOX4 (sc-21860, 1:100 dilution). They were purchased from Santa Cruz Biotechnology. After washing in PBS, sections were further incubated with biotinylated secondary antibodies (1:300 dilution, ABC staining system kit, Santa Cruz, CA, United States), Alexa 594-labeled anti-mouse secondary antibody (at 1:200 dilution, red fluorescence) (Invitrogen, CA, United States) for 60 min at room temperature.

### Dihydroethidium (DHE) Staining

Fluorescent-labeled DHE was used to detect superoxide production in the PVN. Coronal sections (18 mm) were incubated with DHE (0.05 mM) for 30 min at 37°C (Li et al., 2014). Sections containing PVN were then rinsed with PBS (0.01 M) three times and were then observed using a Nikon epifluorescence microscope.

### Western Blotting

The PVN tissue was homogenized in lysis buffer and Western blotting was performed as previously described (Li et al., 2014). The protein concentration was measured and loaded onto an SDS-PAGE gel and then transferred to a polyvinylidene fluoride membrane. The membrane was then incubated overnight at 4°C with the primary antibodies including: Nrf2 (ab31163, 1:500 dilution), Cu/Zn-SOD (ab16831, 1:500 dilution), NOX4 (sc-21860, 1:500 dilution), glutamic acid decarboxylase 67 (GAD67) (sc-7512, 1:500 dilution), TH (sc-25269, 1:1000 dilution) in the PVN, and anti-β-actin (sc-8432, 1:2000 dilution). They were purchased from Santa Cruz Biotechnology. After washing with wash buffer four times for 10 min each time, blots were then incubated for 1 h with secondary antibody (1:5,000 dilution, Santa Cruz Biotechnology) labeled with horseradish peroxidase. Protein loading was controlled by probing all blots with β-actin antibody (Thermo Scientific, United States) and normalizing their protein intensities to that of β-actin. Band densities were visualized with Bio-Rad Chemi Doc XRS System and analyzed using ImageJ (NIH).

### Statistical Analysis

All data were shown as mean ± SEM. Blood pressure data were examined using repeated measures ANOVA. Others were analyzed by one-way ANOVA followed by a *post hoc* Tukey test. The number of marker positive neurons were calculated in three consecutive sections for each rat, and an average value was acquired. *P < 0.05* was considered statistically significant.

## Results

### PVN Infusion of OPC Reduced the SBP in 2K-1C Rats

**FIGURE 1:**
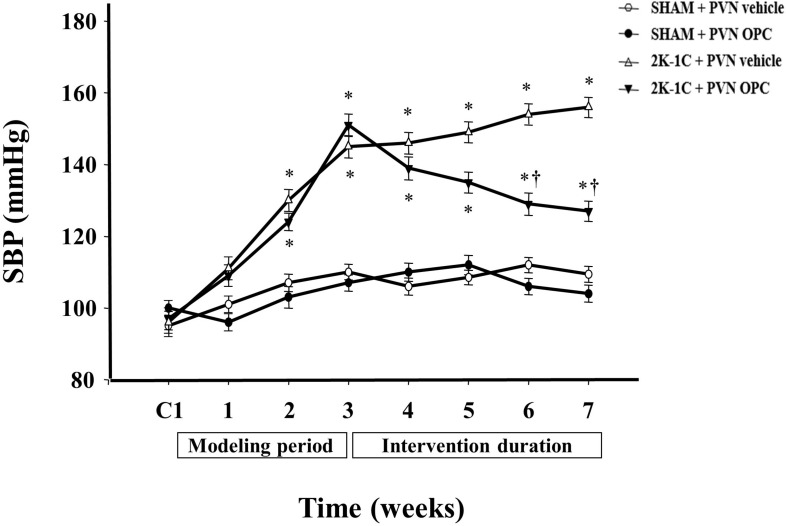
Effects of OPC on SBP. 2K-1C induced significant increase in the SBP, which was reduced by bilateral PVN infusions of OPC. Data are mean ± SEM. ^∗^*P* < 0.05 versus control rats (SHAM + PVN aCSF or SHAM + PVN OPC); †*P* < 0.05 versus 2K-1C + PVN aCSF. *n* = 6 per group. C1, three days before the experiment.

There were no differences of the base blood pressure among all groups (Figure 1). 2K-1C induced a significant increase in SBP compared with the SHAM group, whereas chronic PVN infusion of OPC attenuated the 2K-1C-induced increase in SBP (Figure 1). However, IP treatment with the same doses of OPC had no effect on SBP of the 2K-1C rats.

### PVN Infusion of OPC Influenced NE in the Plasma of 2K-1C Rats

**FIGURE 2:**
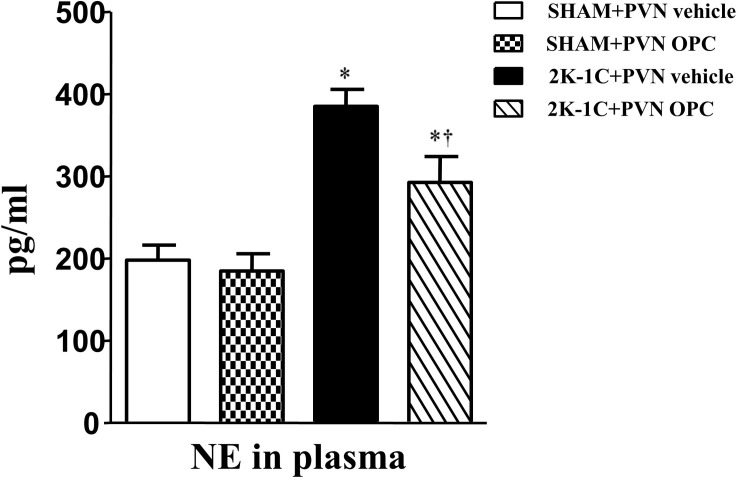
Effects of OPC on levels of NE in the plasma of rats. 2K-1C induced higher level of NE in the plasma than that of SHAM ones, and PVN infusions of OPC alleviated NE elevation in the PVN of the 2K-1C rats. Data are mean ± SEM. ^∗^*P* < 0.05 versus control rats (SHAM + PVN aCSF or SHAM + PVN OPC); †*P* < 0.05 versus 2K-1C + PVN aCSF. *n* = 6 per group.

2K-1C rats had higher plasma level of NE than those of SHAM rats, whereas chronic PVN infusion of OPC for 4 weeks attenuated the 2K-1C-induced increase in plasma NE (Figure 2). However, IP treatment with the same doses of OPC had no effect on NE in the plasma of the 2K-1C rats.

### PVN Infusion of OPC Increased the Expressions of Nrf2 in the PVN of 2K-1C Rats

**FIGURE 3:**
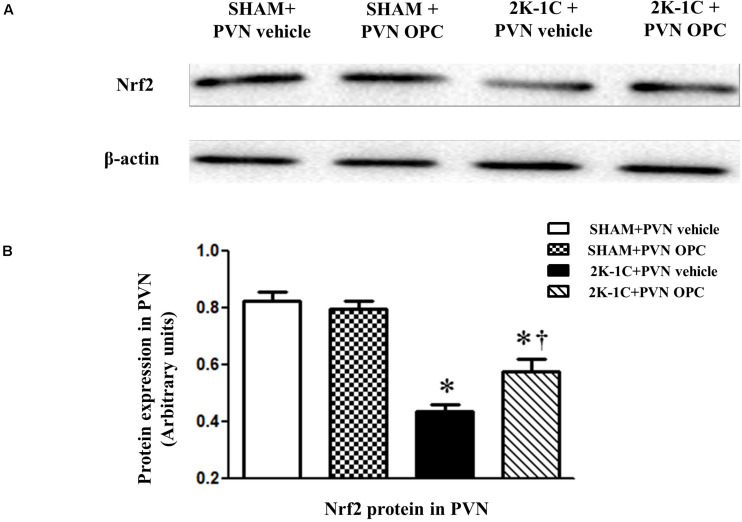
Effects of OPC on protein expression of Nrf2 in the PVN. **(A)** Western blotting results of Nrf2 and β-actin expression; **(B)** Statistical results of Nrf2 expression; Data are normalized to β-actin and shown as mean ± SEM. ^∗^*P* < 0.05 versus control group (SHAM + PVN aCSF or SHAM + PVN OPC); †*P* < 0.05 versus 2K-1C + PVN aCSF. *n* = 6 per group.

Two-kidney one-clip (2K-1C) rats showed lower levels of Nrf2 in the PVN than those of SHAM rats, whereas chronic PVN infusion of OPC for 4 weeks enhanced Nrf2 expression in the PVN of 2K-1C rats (Figure 3). The result indicates that PVN administration of OPC activates Nrf2 pathway in the PVN of 2K-1C rats.

### PVN Infusion of OPC Attenuated Oxidative Stress in the PVN of 2K-1C Rats

**FIGURE 4:**
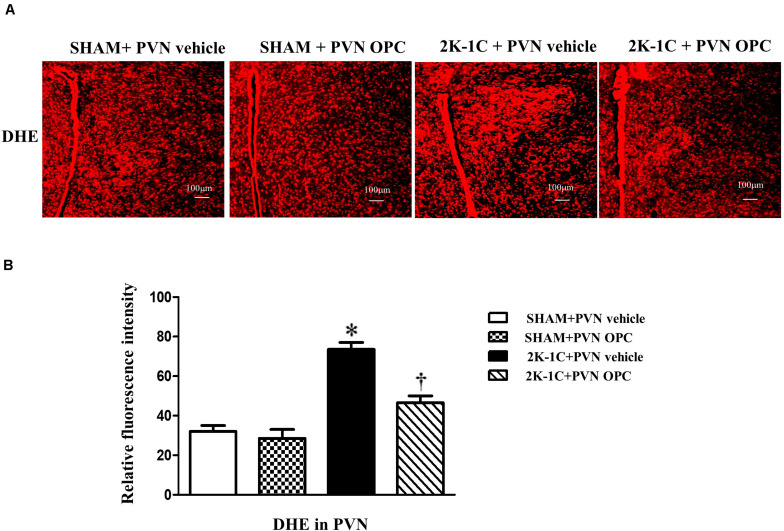
Effects of PVN infusion of OPC on DHE in the PVN of 2K-1C rats. **(A)** The DHE displayed superoxide anion production (red fluorescence) in the PVN; **(B)** Relative fluorescence intensity of DHE in the PVN. Data are mean ± SEM. ^∗^*P* < 0.05 versus control group (SHAM + PVN aCSF or SHAM + PVN OPC); †*P* < 0.05 versus 2K-1C + PVN aCSF. *n* = 6 per group.

The DHE staining was conducted to reflect superoxide anion production. 2K-1C rats revealed higher levels of DHE in the PVN than those of SHAM rats, whereas chronic PVN infusion of OPC for 4 weeks attenuated DHE in the PVN of 2K-1C rats (Figure 4).

### PVN Infusion of OPC Increased the Production of Superoxide Dismutase (SOD) in the PVN of 2K-1C Rats

**FIGURE 5:**
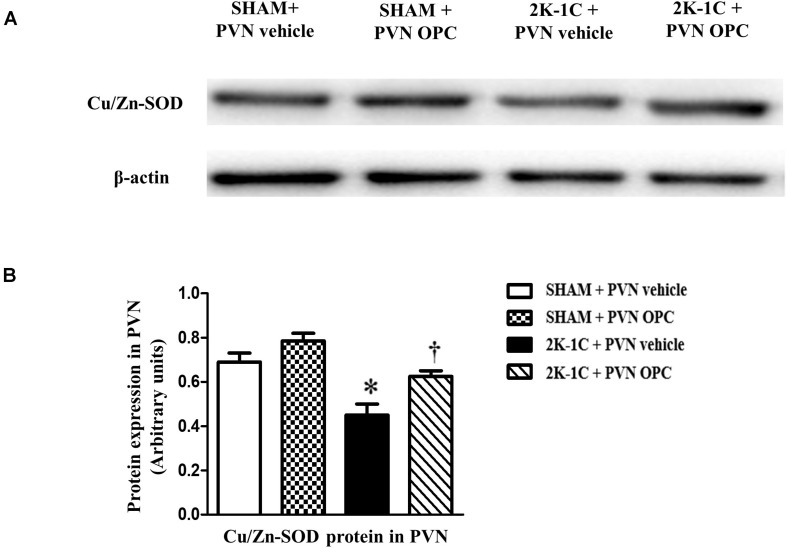
Effects of PVN infusion of OPC on Cu/Zn SOD in the PVN of 2K-1C rats. **(A)** Western blotting results of Cu/Zn-SOD and β-actin expression; **(B)** Statistical results of Cu/Zn-SOD expression; Data are modified by β-actin and shown as mean ± SEM. ^∗^*P* < 0.05 versus control group (SHAM + PVN aCSF or SHAM + PVN OPC); †*P* < 0.05 versus 2K-1C + PVN aCSF. *n* = 6 per group.

The antioxidant enzymes are one part of the cellular defense system against oxidative stress. We measured the expression level of Cu/Zn-SOD with or without OPC administration. Compared with SHAM rats, the level of antioxidant enzyme Cu/Zn-SOD in the PVN was lower in 2K-1C rats (Figure 5). Compared with the control group, 2K-1C-induced inhibitory effects on Cu/Zn-SOD in the PVN were improved by treatment with OPC for 4 weeks (Figure 5). These results indicate that PVN infusion of OPC increased the production of SOD in the PVN of 2K-1C rats.

### PVN Infusion of OPC Inhibited NOX4 in the PVN of 2K-1C Rats

**FIGURE 6:**
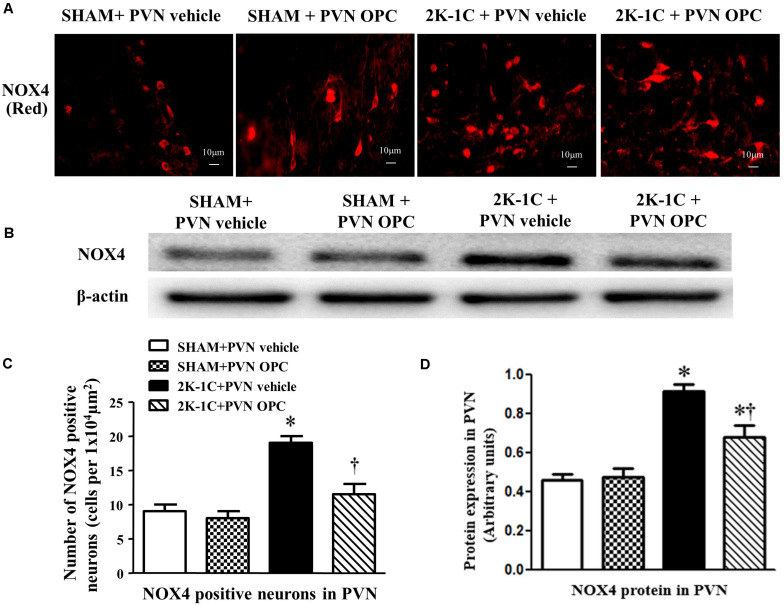
Effects of OPC on NOX4 in the PVN of 2K-1C rats. **(A)** Immunofluorescent staining of NOX4 (red fluorescence) in the PVN; **(B)** The number of NOX4 positive neurons per 1 × 10^4^ μm^2^; **(C)** Western blotting results of NOX4; **(D)** Statistical results of NOX4 expression. Data are mean ± SEM. ^∗^*P* < 0.05 versus control group (SHAM + PVN aCSF or SHAM + PVN OPC); †*P* < 0.05 versus 2K-1C + PVN aCSF. *n* = 6/group.

The NAD(P)H oxidases subunit NOX4 was observed by immunofluorescence staining, and the protein level of NOX4 was detected by Western blotting. Compared with SHAM rats, 2K-1C rats had higher level of NOX4 in the PVN (Figure 6). Compared with the control group, bilateral PVN infusion of OPC for 4 weeks decreased NOX4 expressions in the PVN of 2K-1C rats (Figure 6).

### PVN Infusion of OPC Influenced the Expression of Cytokines in the PVN of 2K-1C Rats

**FIGURE 7:**
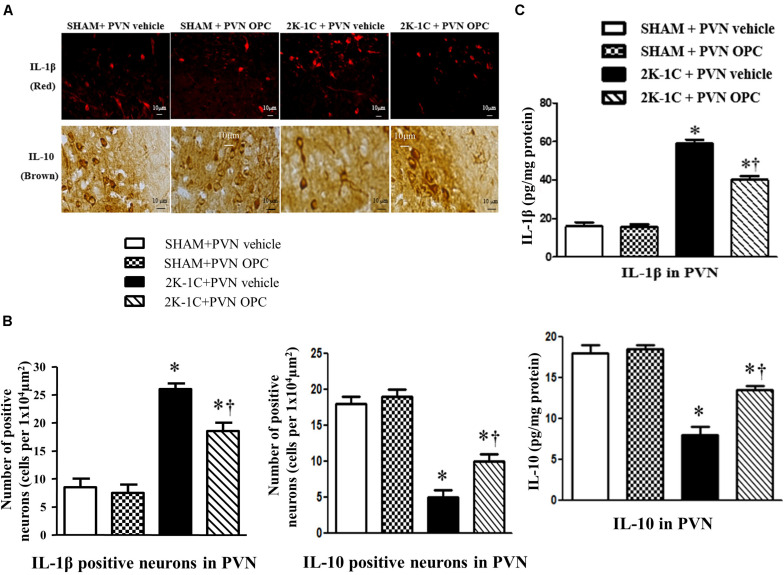
Effects of PVN infusion of OPC on IL-10 and IL-1β in the PVN of 2K-1C rats. **(A)** Immunofluorescent staining of IL-1β (red fluorescence, × 400) and Immunohistochemical staining of IL-10 (brown) in the PVN; **(B)** The number of IL-1β and IL-10 positive neurons per 1 × 10^4^ μm^2^; **(C)** Top: Roles of OPC on the expression of IL-1β; Bottom: Effects of OPC on the expression of IL-10. Data are mean ± SEM. *P < 0.05 versus control rats (SHAM + PVN aCSF or SHAM + PVN OPC); †*P* < 0.05 versus 2K-1C + PVN aCSF. *n* = 6 per group.

2K-1C rats showed higher PVN level of IL-1β and lower PVN level of IL-10 than those of SHAM rats, whereas chronic PVN infusion of OPC for 4 weeks attenuated the 2K-1C-induced increase in IL-1β and the 2K-1C-induced decrease in IL-10 in the PVN of 2K-1C rats (Figure 7). These results indicate that PVN infusion of OPC improved the 2K-1C-induced imbalance of pro-inflammatory cytokines and anti-inflammatory cytokines in the PVN.

### PVN Infusion of OPC Suppressed Neuronal Activity in the PVN of 2K-1C Rats

**FIGURE 8:**
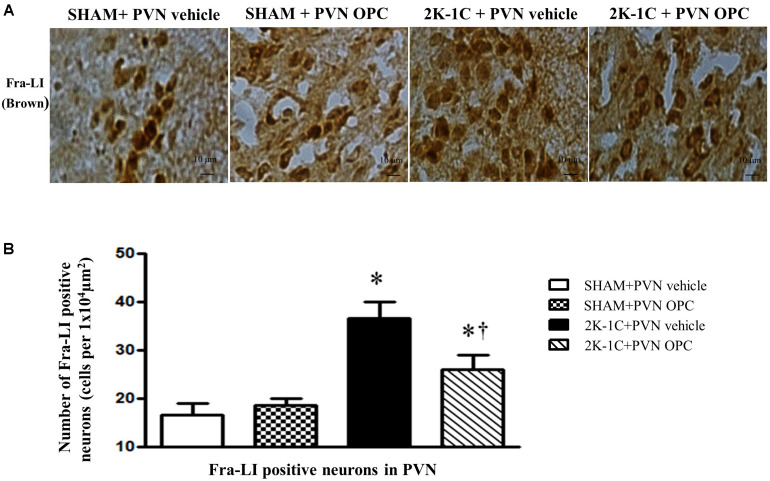
Effects of PVN infusion of OPC on neuronal activity in 2K-1C rats. **(A)** Immunohistochemical analysis about the expression of Fra-LI in the PVN; **(B)** The number of Fra-LI positive neurons per 1 × 10^4^ μm^2^. Data are mean ± SEM. ^∗^*P* < 0.05 versus control group (SHAM + PVN aCSF or SHAM + PVN OPC); †*P* < 0.05 versus 2K-1C + PVN aCSF. *n* = 6 per group.

To observe the effect of PVN infusion of OPC on neuronal activity, we examined the expression of Fra-LI (a marker of chronic neuronal activation). Immunohistochemical staining revealed that 2K-1C rats had more Fra-LI positive neurons in PVN than those of SHAM rats, whereas chronic PVN infusion of OPC for 4 weeks attenuated the 2K-1C-induced increase in Fra-LI positive neurons in the PVN (Figure 8).

### PVN Infusion of OPC Influenced TH and GAD67 in the PVN of 2K-1C Rats

**FIGURE 9:**
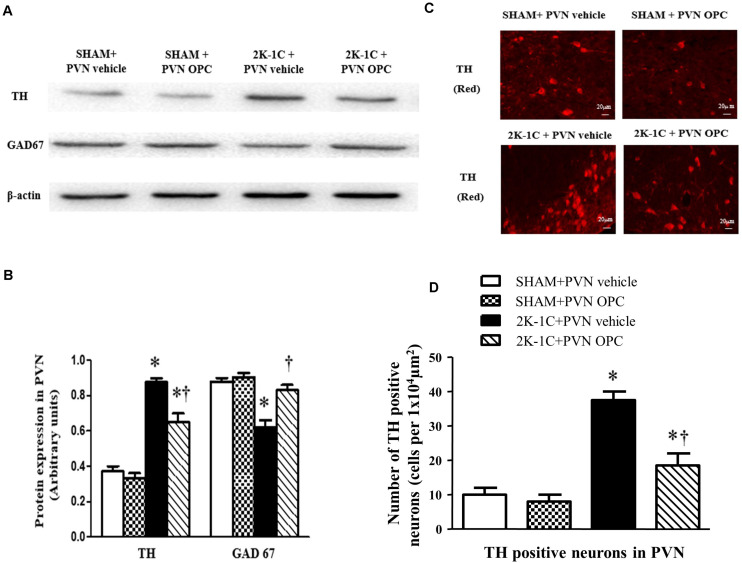
Effects of PVN infusion of OPC on expression of TH and GAD67 in the PVN. **(A)** Western blotting results of TH and GAD67 expressions in the PVN; **(B)** Statistical results of TH and GAD67 expression in the PVN; **(C)** Immunofluorescence staining of TH (red fluorescence) in the PVN; **(D)** the number of TH positive neurons per 1 × 10^4^ μm^2^. Data are normalized to β-actin and shown as mean ± SEM. ^∗^*P* < 0.05 versus control group (SHAM + PVN aCSF or SHAM + PVN OPC); †*P* < 0.05 versus 2K-1C + PVN aCSF. *n* = 6 per group.

Immunofluorescence and Western blotting revealed that 2K-1C rats had more expressions of TH and less expressions of GAD67 in the PVN (Figure 9). PVN infusion of OPC for 4 weeks decreased TH expressions and increased GAD67 expressions in the PVN of 2K-1C rats (Figure 9).

## Discussion

Our findings in the present study are as follows: (1) In 2K-1C-induced hypertensive rats, PVN administration of OPC regulates the sympathetic excitability and reduces blood pressure; (2) In 2K-1C-induced hypertensive rats, PVN administration of OPC may activate Nrf2 pathway to remove oxidative stress and restore the balance of inflammatory and anti-inflammatory cytokines; (3) In 2K-1C-induced hypertensive rats, PVN administration of OPC suppresses neuronal activity and restores the balance between the excitatory neurotransmitter and inhibitory neurotransmitter in PVN.

Proanthocyanidins have been demonstrated to have positive effects on various metabolic disorders such as inflammation, obesity, diabetes and insulin resistance. They reduce the plasma levels of atherogenic apolipoprotein B-triglyceride-rich lipoproteins and LDL-cholesterol but increase antiatherogenic HDL-cholesterol (Bladé et al., 2010).

Paraventricular nucleus (PVN) is one of the key areas of cardiovascular regulation and is also the main site of sympathetic pre-neurons in the brain. It is closely related to the occurrence and development of hypertension and plays an important role in maintaining cardiovascular activity. Studies have shown that the occurrence of hypertension is often accompanied by the activation of sympathetic nerves, and the degree of activity is positively correlated with the level of blood pressure.

We used the 2K-1C rat model, which has provided many insights into the pathogenesis of renovascular hypertension. Then we measured the blood pressure changes of each group of rats. The hypertensive rat model constructed by the 2K-1C method has stable blood pressure, high reliability, and convenient and feasible features. Moreover, the success rate of preparing the model is high, and the mortality rate is low (about 5 to 10%). It has been pointed out that the blood pressure is measured regularly every week after surgery. When the SBP of the rats is greater than 140 mmHg or is 20 mmHg higher than the that before the operation, the 2K-1C hypertension model is successfully prepared.

The results of this experiment showed that compared with the sham-operated rats, the blood pressure of the model group was significantly increased. The SBP maintained at 150 mmHg after three weeks, which was about 40 mmHg higher than that before surgery, indicating that the model was successfully prepared. Other studies reported the same. In this experiment, after chronic administration of OPC in the PVN, the blood pressure of the model group was significantly reduced, and the blood pressure gradually stabilized at about 120 mmHg at the 4th week of administration. In this study, tail-cuff method was used to measure blood pressure. It requires handling and restraint during measurement, which might affect the blood pressure and undermining reliability of the results (Wilde et al., 2017). The SBP measured with the catheters was consistent with the tail-cuff occlusion (Ibrahim et al., 2006; Feng et al., 2008; Fritz and Rinaldi, 2008). At the end of the experiment, the BP measured with the catheters in this study was consistent with tail-cuff occlusion.

Oxidative stress occurs when ROS accumulate in the body and the imbalance of oxygen free radical production and elimination causes oxidative damage. ROS is a general term for a class of oxygen-containing and active substances. NADPH oxidases, lipoxygenase, xanthine oxidase, mitochondrial respiratory chain and NO synthase are involved in the production of ROS. SOD and reduced glutathione are involved in the clearance of ROS. This study found that the production of ROS in the PVN region of hypertensive rats increased, which can enhance sympathetic excitability and promote the progress of hypertension. Chronic administration of tempol, one of oxygen free radical scavengers in the lateral ventricle can attenuate the above changes (Kang et al., 2019). Chronic administration of Nrf2 activator tBHQ via PVN can reduce oxidative stress and inflammatory response in rat PVN region, improve neurotransmitter imbalance in paraventricular nucleus, and reduce sympathetic excitability and blood pressure (Bai et al., 2017). OPC has antioxidant biological effects and can lower blood pressure, but its central mechanism of action in the PVN region is still unclear. The results of this experiment showed that in the hypertensive rats the expression of Nrf2 protein in the PVN region of hypertensive rats decreased, the expression of ROS and NOX4 were elevated significantly, and the expression of Cu-Zn SOD protein decreased. After OPC were administered, the expression of Nrf2 protein in the PVN region was increased, the expression of ROS and NOX4 was significantly decreased, and the expression of Cu-Zn SOD protein was significantly increased. Therefore, OPC may antagonize oxidative damage through the Nrf2 pathway to reduce ROS production and lower blood pressure. However, there is a limitation in this study that only high magnification images were shown.

Experiments showed that the expression of IL-1β in the PVN region of spontaneously hypertensive rats was elevated, and the expression of anti-inflammatory cytokine IL-10 decreased, indicating that hypertension is accompanied by inflammation. Studies have also found that ROS interacted with inflammatory cytokines to increase sympathetic activity and increase blood pressure (Haspula and Clark, 2018). We also found that the expression of ROS in the PVN of the hypertensive model group was increased. After administration of OPC, the expression of ROS in the PVN region was decreased, the expression of IL-1β was significantly decreased, and the expression of IL-10 was significantly increased. These results indicate that PVN infusion of OPC improved the 2K-1C-induced imbalance of pro-inflammatory cytokines and anti-inflammatory cytokines in the PVN.

Studies have shown that central excitatory and inhibitory neurotransmitters are imbalanced in hypertensive disease. Glutamate and norepinephrine in the PVN region act as excitatory neurotransmitters to enhance sympathetic nerve activity, while gamma-aminobutyric acid acts as an inhibitory neurotransmitter to attenuate sympathetic activity. TH is the norepinephrine synthesis rate-limiting enzyme. It is responsible for the transformation of amino acid L-tyrosine to L-3,4-dihydroxyphenylalanine, which is the precursor for the neurotransmitters (NE and adrenaline). As TH is present in the central nervous system, we used the TH to represent the excitatory activity of neurons. GABA is a neurotransmitter in the brain. GAD is the rate-limiting enzyme of gamma-aminobutyric acid synthesis that catalyzes the decarboxylation of glutamate to GABA and CO_2_. Thus, we used GAD67, an isoform of GAD to represent the inhibitory activity of neurons. The immunofluorescence and Western blotting results of this experiment showed that the expression of TH in the PVN region was increased in the hypertensive rat model. The expression level of GAD67 decreased in the hypertensive rats. After chronic administration of OPC through the PVN, the expression level of TH decreased and the expression level of GAD67 increased. This suggests that OPC can improve the imbalance of excitatory neurotransmitters and inhibitory neurotransmitters, reduce sympathetic activity, and lower blood pressure.

## Conclusion

The rats prepared by the 2K-1C method showed elevated blood pressure, increased sympathetic nerve activity, increased production of ROS, high expression of pro-inflammatory cytokines, low expression of anti-inflammatory factors, and excitatory neurotransmitters and inhibitory neurotransmitters are out of balance in the PVN. After chronic administration of OPC in the PVN, the oxidative stress and inflammatory cytokine are regulated, possibly through the Nrf2 pathway. OPC also improves neurotransmitter imbalance, and thereby reduces sympathetic activity and lowers blood pressure.

## Data Availability Statement

The original contributions presented in the study are included in the article/supplementary material, further inquiries can be directed to the corresponding author/s.

## Ethics Statement

The animal study was reviewed and approved by the Institutional Animal Care and Use Committee (IACUC) of Xi’an Jiaotong University.

## Author Contributions

Y-MK and X-JY designed the study. G-RX, L-YF, and K-LL performed all experiments. Y-MK and X-JY also performed the data analysis and drafted the manuscript. Y-MK, G-RX, and X-JL participated in data analysis. Y-MK, X-JY, KK, X-JL, JQ, Q-YY, T-TM, Y-JS, and YL critically revised the manuscript. All authors reviewed the final manuscript.

## Conflict of Interest

The authors declare that the research was conducted in the absence of any commercial or financial relationships that could be construed as a potential conflict of interest.

## Abbreviations

2K-1C: two-kidney, one-clip
aCSF: artificial cerebrospinal fluid
GAD: glutamic acid decarboxylase
IL-1 β: interleukin-1 beta
IL-10: interleukin-10
NADPH: nicotinamide adenine dinucleotide phosphate
NE: norepinephrine
Nrf2: nuclear factor erythroid-2-related factor 2
OPC: oligomeric proantho cyanidins
PBS: phosphate-buffered solution
PVN: hypothalamic paraventricular nucleus
ROS: reactive oxygen species
SBP: systolic blood pressure
SHAM: sham-operated
SOD: Superoxide Dismutase
TH: tyrosine hydroxylase.
